# Reflection paper on copyright, patient-reported outcome instruments and their translations

**DOI:** 10.1186/s12955-018-1050-4

**Published:** 2018-12-05

**Authors:** Caroline Anfray, Benjamin Arnold, Mona Martin, Sonya Eremenco, Donald L. Patrick, Katrin Conway, Catherine Acquadro, on behalf of the ISOQOL Translation and Cultural Special Interest Group (TCA-SIG)

**Affiliations:** 1 Mapi Research Trust, 27 rue de la Villette, 69003 Lyon, France; 2 FACITtrans, Elmhurst, IL USA; 3 Health Research Associates, Mountlake Terrace, WA USA; 4 PRO Consortium, Critical Path Institute, Tucson, AZ USA; 5 University of Washington, Seattle, WA USA; 6 Mapi, an ICON plc Company, Lyon, France

**Keywords:** Patient-reported outcomes, Clinical outcome assessments, PRO instruments, Copyright, Intellectual property, Translation

## Abstract

With the growth of patient-reported outcome (PRO) measurement, questions arise regarding how copyright protection applies to PRO instruments in general and to their translations in particular. The main objectives of this reflection paper are: 1) to help authors of PRO instruments understand basic rules of intellectual property and copyright that protect the integrity of their instruments and derivatives; and 2) to provide recommendations to authors and users of PRO instruments to prevent misuse or abuse.

National laws on intellectual property (IP) and the international Berne Convention fully apply to PRO instruments since they are creations of the mind. Therefore, the copyright holder / owner / claimant of a PRO instrument, i.e., the person or legal entity who owns the copyright of the instrument, is granted exclusive rights that are divided into two main categories: moral and economic rights. Moral rights are: 1) the right of attribution (or right of paternity), i.e., the right to claim authorship of the work, 2) the right against false attribution, and 3) the right of integrity, i.e., the right to object to any mutilation, deformation or modification of the work. Economic rights represent the exclusive rights of the author to make or authorize reproduction, development of derivative works, distribution and communication to the public. In other words, the PRO instrument’s copyright holder controls access (distribution, reproduction), and authorizes all derivative works, i.e., adaptations (e.g., electronic formats), modifications (e.g., shorter versions), and translations. Hence, the access to and use of an original PRO instrument and its derivatives in any kind of research should always be associated with the identification of its copyright holder. However, in some cases, this identification may be challenging, in particular when copyright ownership is not clearly defined. To prevent ownership conflicts as well as misuse or abuse of PRO instruments, the ISOQOL Translation and Cultural Adaptation Special Interest Group (TCA-SIG) provides recommendations to authors of PRO instruments and their users. In particular, the TCA-SIG recommends that the ownership of PRO instruments and their derivatives should be defined from the beginning (i.e., from the development of the instrument) and along the life cycle of the instrument between all parties involved. These recommendations apply not only to PRO instruments but also to all the other clinical outcome assessments (COAs), since they are also creations of the mind.

## Introduction

The overall objectives of the Translation and Cultural Adaptation Special Interest Group (TCA-SIG) are to identify and advance research practices and outcomes of translation and cultural adaptation of patient-reported outcome (PRO) instruments; to provide an evidence database on translation and cultural adaptation of PRO instruments; and to promote visibility of cross-cultural issues in the development and use of PRO instruments in ISOQOL. The copyright subgroup of the TCA-SIG has focused its activities on issues linked to the copyright of original instruments and their derivatives such as translations. The main objectives of this reflection paper are: 1) to help authors of PRO instruments understand basic rules of intellectual property and copyright in order to protect the integrity of their instruments and derivatives; and 2) to provide recommendations to authors and users of PRO instruments in order to prevent any misuse or abuse.

## Copyright: definition and legal aspects

According to the US Copyright Act [1], copyright exists in all original work of authorship fixed in a tangible medium of expression. “Original work” means that the work is not received from others nor is copied from or based upon the work of others and possesses at least a small amount of creativity.

Copyright [1, 2] is a group of exclusive rights that provides the owner of the copyrighted work the exclusive right to (i) reproduce the work in copies; (ii) prepare derivative works (a “derivative work” is a work “*based upon one or more preexisting works, such as a translation […], abridgment, condensation, or any other form in which a work may be recast, transformed or adapted*” [1]); (iii) distribute copies of the work to the public by sale or other transfer of ownership, or by rental, lease, or lending;(iv) display the copyrighted work publicly, and (v) communicate the work to the public.

Copyright is regulated by national copyright laws and, at an international level, by the Berne Convention for the Protection of Literary and Artistic Works as revised in Paris on July 24, 1971 (hereinafter referred to as the “Berne Convention”). The Berne Convention was implemented on a country-by-country basis when a member country ratified it and thereby officially became a party to it. For example, the USA ratified the Berne Convention by enacting the Berne Convention Implementation Act of 1988 (H.R. 4262) on October 31, 1988, thereby implementing and joining the Berne Convention on March 1, 1989. Other countries such as Belgium, France, Germany, Italy, Spain, Switzerland and the United Kingdom implemented it on December 5, 1887. A listing of contracting parties to the Berne Convention and the respective dates when it was implemented in a country can be found in the World Intellectual Property Organization (WIPO) database (http://www.wipo.int/treaties/en/ShowResults.jsp?treaty_id=15).

The Berne Convention aims to protect the rights of authors in their literary, scientific and artistic works at an international level [2] in a manner as effective and uniform as possible. It stipulates that copyright is automatic (it belongs to the “creator” of the work - no registration is needed) and gives the copyright holder minimum exclusive rights that are divided into two main categories: moral rights and economic rights.

Moral rights are: 1) the right to claim authorship of the work (right of attribution or right of paternity), 2) the right against false attribution, and 3) the right of integrity, i.e., the right to object to any mutilation, deformation or modification of the work or any derogatory treatment, which constitutes any act in relation to a work that is in any manner harmful to the author’s honor or reputation. Economic rights represent the exclusive rights of the author to make commercial gain from the exploitation of his work, and to authorize reproduction, distribution, preparation of derivative works, and communication to the public of their original work. Moral rights, unlike economic rights, protect the non-economic interests and cannot be transferred, given away, sold or otherwise disposed of. However, the conditions of use of an instrument and associated fees are decided solely by the copyright holder. Even if copyrighted, the copyright holder may decide that the work can be accessed free of charge, or payable only by a category of users.

Copyright is subject to a time limit. In general, copyright lasts for the life of the author and expires 50 or 70 years after his / her death (depending on national laws).

If a work is not protected by copyright or any other proprietary right (such as trademark or patent), then it is considered being in the “public domain”. This can be because the copyright has expired or because the creator of the work gave up its copyright and decided to let the public freely use, modify, and adapt its work.

## How do laws about intellectual property apply to patient-reported outcome (PRO) instruments?

PRO instruments provide evidence of health status reported by the patient. They are usually employed to collect data during clinical trials and to provide information about the patient’s view of treatment effect [3]. Since PRO instruments are a means to collect health data, the integrity of their content (instructions, items and response categories) as well as their measurement properties are key features for regulatory authorities such as the Food and Drug Administration and the European Medicines Agency. Guidelines from these agencies require that any change to content be documented and justified [3, 4], and modifications often require additional evidence to support content validity of the modified instrument.

PRO instruments constitute literary works protected by copyright since they are original creations of the mind, created in a fixed, tangible form of expression, independent of whether they are categorized as scientific works. Hence, national laws on IP and, at an international level, the Berne Convention fully apply to PRO instruments. Therefore, the copyright claimant of a PRO instrument owns the moral and economic rights of the work, as described in the above section of this article. Through the moral rights, the integrity of the PRO instruments is protected, which is essential for regulatory reasons, as stated earlier.

However, if the copyright holder is not clearly mentioned on the instrument, its identification can be challenging and may lead to infringement, and misuse or abuse of the instrument [5]. There are situations when more than one entity (i.e., researcher, sponsor or university) may qualify for copyright ownership [6, 7]. Furthermore, at the time of the publication or communication of the instrument, the question of copyright ownership can be raised again, as the economic rights might be transferred partially or fully to the publishers or the journal.

A clear identification of the copyright holder (through copyright notice) directly on the instrument is essential, as well as the drafting of a contract from the development phase of the instrument to the derivative phase, to avoid any future question or dispute of ownership.

## Copyright of translations of PRO instruments

### Laws and existing recommendations

As stated earlier, only the copyright owner of the original instrument can authorize a translation to be developed. It is the responsibility of the entity carrying out such translation, thereby constituting a derivative work, to firstly obtain the necessary permission from the copyright owner of the original, otherwise, the translation would be considered as a copyright infringement. It is also the translator’s (or translation agency’s) responsibility to make sure that the translation is faithful to the original. If the translation is not true to the original, or alters or modifies its very sense, it will constitute a violation of a moral right of the author of the original.

Translators, as “author” of their translations can be protected by copyright laws. For instance, in France, Article L. 112–3 of the French “Code de la Propriété Intellectuelle” (Intellectual Property Code) [8] states that the authors of translations benefit from the same rights given to authors, without prejudice to the rights of the original author (on the condition, of course, that the copyright holder of the original work gave its authorization). In the USA [1] for example, translations may also fit into the work “made for hire” category, i.e., a work prepared by an employee within the scope of his or her employment; or a work specially ordered or commissioned, if the parties expressly agree in a written instrument signed by them that the work shall be considered a work made for hire. In this case, the commissioning party is considered the legal author. This is an exception to the general rule that the person who actually creates a work is the legally recognized author of that work. On an international level, UNESCO has adopted a recommendation [9], which specifies that: *“Member States should accord to translators, in respect of their translations, the protection accorded to authors under the provisions of the international copyright conventions to which they are party and/or under their national laws, but without prejudice to the rights of the authors of the original works translated.”*

If the translator received proper authorization to develop the translation, and in the absence of any statement from the copyright holder of the original questionnaire that he/she owns copyright on the translations of his questionnaire, or the absence of a written contract between the copyright holder and the translator, the translator may claim copyright on the translation.

One of the risks of the copyright holder not properly tracking and coordinating the library of translations of her/his instrument for use in international studies is the multiplication of “same language” translations (e.g., several Castilian Spanish versions of the same original). In such a case, it would be almost impossible to identify the “right” translation. In the context of international clinical trials, this situation can be very problematic for users. In other words, a copyright claimant of the original should not authorize another suitable translation in the identical language of an existing translation.

#### Example of controversy

The controversy around the Greek version of the standardized version of the Asthma Quality of Life Questionnaire [AQLQ(s)] is an example of the difficulties inherent in copyright faced by authors and users of PRO instruments [10]. The 18-item Greek version [11] was considered an unauthorized modified version, derived from the copyrighted 32-item Greek version of the AQLQ(S) [12]. In this case, the translator of this 18-item Greek version did not ask permission from the copyright holder to distribute the translation. Hence, this version can no longer be used under the name “AQLQ”.

The complexities of the case, however, have had a positive outcome which is best described by Elizabeth Juniper’s reaction as author of the AQLQ: *“the (..) paper has (…) started an important discussion about the right to modify, translate or adapt questionnaires without the permission of the copyright holder”* [12]. Preserving the integrity of the original instrument and its translations is one of the important reasons for authors to exercise their rights [5, 6]. Revicki and Schwartz emphasized this crucial issue in their editorial [13]: *“the maintenance of the scientific integrity of the copyrighted instrument […] will ensure researchers and readers of scientific journals that the study used the correct version and that there is evidence supporting the psychometric qualities of the instrument.”*

To conclude, the reasons for controlling the copyright of the translations are the following: (1) to preserve the integrity of the translated instrument, (2) to control its proper use and avoid multiplication of versions, and (3) to provide easy access to the users. If the true chain of custody for copyright ownership of the translations is not known, then it is difficult to access and use them without clear risk or copyright infringement*.*

## Special considerations with shared intellectual property: PROMIS® (see special acknowledgement)

Certain types of projects such as item banking initiatives like the Patient Reported Outcomes Measurement Information System (PROMIS**®**) effort handle intellectual property in a way that explicitly encourages free use of copyright-protected materials. Copyright ownership is distributed widely across PROMIS**®** investigators and colleagues in the measurement community who have agreed to allow the PROMIS**®** Health Organization (PHO), a not-for-profit charitable organization, to manage and distribute the tools over time on behalf of the United States National Institutes of Health (NIH). The PROMIS**®** item banks comprise more than 1000 questions, many of which derive from or resemble questions that exist in other questionnaires. In cases of derivative items used in PROMIS**®**, initial authors have agreed to allow the PHO to license and distribute the collective intellectual property (IP). At the same time, the US NIH owns the PROMIS**®** Trademark and thereby has a role in the maintenance of the PROMIS**®** quality standard, a responsibility to be entrusted to the PHO. Data collected with PROMIS**®** instruments are considered the property of the researcher. Original items in the PROMIS**®** item banks have been provided and shared with PROMIS**®** by their respective IP holders in order to advance the larger scientific initiative of outcomes measurement. The PHO serves as an umbrella organization, monitoring and ensuring that any parties who wish to develop any derivative products of the items (such as translations or modifications) contractually agree that the IP of any derivation is held and managed by the PHO on behalf of the common good. This centralized control mechanism, though intricate and challenging to establish, serves to protect all the interests of the respective PROMIS**®** parties as well as those who shared their IP with PROMIS**®** investigators.

In the same line of thought, early copyright rules have been developed for consortia-developed instruments [14].

## Final recommendations

To prevent conflicts, misuse and abuse of PRO instruments, we propose several recommendations to authors and users of PRO instruments:

- Recommendations for authors
  1. Protect your copyright
    In countries which have ratified the Berne Convention, PRO instruments are protected “de facto” by law, which is a significant advancement in copyright protection. In these countries, copyright registration is not mandatory, but is desirable, specifically in case of potential copyright infringement. A posteriori proof of ownership is always difficult. Although it seems contradictory, experience has shown that registering copyright with local copyright agencies or by any other means, is the safest way to prove the author’s ownership and anteriority on the instrument. Authors are therefore advised to register their work in their country of residence with the local copyright agency or seek advice to private practices or companies specialized in copyright protection, in order to avoid contestation of ownership and to protect the integrity of the instrument. Registration establishes a public record of the copyright claim.
  2. Write a contract
    The ownership of PRO instruments and their derivatives should be defined from the very beginning (at the development phase of the instrument) between all parties involved and stated in a written agreement. Each step in the questionnaire’s life should be anticipated with copyright ownership in mind.
  3. Be careful when you publish
    You should not publish the instrument in extenso (in its entirety) in a scientific journal. If possible, only publish extracts of the instrument. If not, the contract between the author and the publisher must clearly state that the copyright on the instrument itself vests in the author.
  4. Establish rules
    Anticipate the conditions of use of your instrument (i.e., license agreement, translation agreement, use of data, fees, etc.) and have them stated in writing. Of note, copyright should not be seen as an obstacle to easy access and use, i.e., royalty fees are neither mandatory nor systematic to get access to a copyrighted work; it can be free.
  5. Make your copyright notice visible
    Even if the copyright notice is not a requirement, it is beneficial to remind the reader or user that the work is protected to limit any violation of copyright. A copyright notice should contain the word “copyright,” a “c” in a circle (©), the date of publication, and the name of the owner of all the copyright rights in the published work.
  6. Privilege a unique copyright holder for original work and derivatives
    Copyright of a PRO instrument and its derivatives, including but not limited to translations and electronic versions, should be owned by a unique copyright holder, ideally the original author, to harmonize and facilitate conditions of access and use.
  7. Centralize distribution
    The distribution should be centralized, ideally by the original author, to (a) facilitate access to questionnaires, (b) maintain reliable information about them as per regulatory requirements and (c) control their use, e.g., specifically for translations where multiplication of translations for a same language should be avoided.
  8. Get counsel
    It may seem expensive and unnecessary, but getting legal counsel for the legal protection of your instruments and guidance regarding the management and distribution of your instruments could save you many problems later. A legal advisor specialized in intellectual property will help you register your copyright and draft the appropriate agreements. Specialized PRO organizations may also assist in the process as they bridge the gap between the PRO good practices and the copyright laws.
  9. Legacy PRO Instruments
    Following these recommendations and setting-up clear intellectual property on a new PRO instrument during its development is key. However, for legacy instruments that were developed without such considerations, it is the author’s responsibility to clarify the copyright situation by conducting due diligence. For this purpose, the author shall contact all the parties involved in the development and publication of the instrument (universities, hospitals, commercial companies, co-authors, publisher, etc.), to engage in discussions and decide the best way to protect the instrument’s integrity, by consolidating the intellectual property rights in one person (whether natural or legal). Further to these discussions, a contract between the parties involved should be drawn up to settle the management of the copyright of the instrument. Once the due diligence has been performed and the situation regarding the intellectual property cleared up the author may register the copyright on the instrument. Again, some PRO organizations are specialized in this type of due diligence and copyright management and can help the author to perform it.
- Recommendations for users
  1. Respect the copyright
    The unauthorized use of copyrighted works constitutes an infringement of copyright. It means you CANNOT reproduce, distribute, display, or create derivative works without first obtaining proper permission. Furthermore, the fact that there is no copyright notice on an instrument does not mean it is not protected. Therefore, copyright holders must always be looked for and conditions of access of PRO instruments must always be checked prior use with the authors of the instruments.
    When there is no copyright notice, the user must however ensure that it is allowed to use the instrument. If the author has not performed the due diligence above, it will be the user’s responsibility to conduct it, by contacting the persons involved in the development of the instrument. For this purpose, the user may also be assisted by specialized organizations who have good knowledge in the field.
  2. Write a contract
    License/user agreements with copyright holders should be established in written form.
  3. Anticipate
    If you want to use a specific instrument, you must anticipate that it may take time to find the copyright holder and to set-up a license agreement.

These recommendations should apply not only to PRO instruments but also to all the other clinical outcome assessments (COAs), i.e., clinician-reported outcomes (ClinROs) instruments, observer-reported outcomes (ObsROs) instruments and performance outcomes (PerfOs) instruments, since they are developed based on the same scientific principles, and are creations of the mind.

## Conclusion

Copyright and intellectual property of PRO instruments and their derivatives can be complex, yet simple rules should apply. As copyright holders, authors of the original instruments should play a central and crucial role in how their questionnaires are used. They should be the cornerstone on which any request for use, modification, adaptation and translation should be based. Anticipation of copyright ownership at every stage of the instrument’s life cycle (i.e., development, communication, derivatives) may contribute to better use and acceptability of the questionnaires, as well as recognition by the scientific community.

## Abbreviations

AQLQ(s): Standardized version of the Asthma Quality of Life Questionnaire
COA: Clinical outcome assessment
IP: Intellectual property
ISOQOL: International Society for Quality of Life Research
NIH: National Institutes of Health
PHO: PROMIS® Health Organization
PRO: Patient-reported outcome
PROMIS®: Patient-Reported Outcomes Measurement Information System
TCA-SIG: Translation and cultural special interest group
UNESCO: United Nations Educational, Scientific and Cultural Organization
WIPO: World Intellectual Property Organization
